# Maternal 25-Hydroxyvitamin D Deficiency Promoted Metabolic Syndrome and Downregulated Nrf2/CBR1 Pathway in Offspring

**DOI:** 10.3389/fphar.2020.00097

**Published:** 2020-02-28

**Authors:** Jianqiong Zheng, Xiaohui Liu, Bingbing Zheng, Zhenzhen Zheng, Hongping Zhang, Jiayong Zheng, Congcong Sun, Haiying Chen, Jie Yang, Zuo Wang, Meimei Lin, Jingjing Chen, Qingdiao Zhou, Zhi Zheng, Xiaoming Xu, Hao Ying

**Affiliations:** ^1^ Department of Obstetrics, Shanghai First Maternity and Infant Hospital, Tongji University School of Medicine, Shanghai, China; ^2^ Department of Obstetrics and Gynecology, Wenzhou People's Hospital, the Third Clinical Institute Affiliated to Wenzhou Medical University, Wenzhou, China; ^3^ Department of Wenzhou Key Laboratory of Gynecology and Obstetrics, Wenzhou People's Hospital, The Third Clinical Institute Affiliated to Wenzhou Medical University, Wenzhou, China

**Keywords:** maternal, 25-hydroxyvitamin D deficiency, metabolic syndrome, Nrf2/CBR1 pathway, offspring

## Abstract

Metabolic syndrome is a disorder of energy use and storage, which is characterized by central obesity, dyslipidemia, and raised blood pressure and blood sugar levels. Maternal 25-hydroxyvitamin D deﬁciency is known to cause metabolic changes, chronic disease, and increased adiposity in adulthood. However, the underlying mechanism of induced metabolic syndrome (MetS) in the offspring in vitamin D deficient pregnant mothers remains unclear. We identified that maternal 25-hydroxyvitamin D deficiency enhances oxidative stress, which leads to the development of MetS in the mother and her offspring. Further, immunohistochemical, Western blotting, and qRT-PCR analyses revealed that maternal 25-hydroxyvitamin D deficiency inhibited the activation of the Nrf2/carbonyl reductase 1 (CBR1) pathway in maternal placenta, liver, and pancreas, as well as the offspring's liver and pancreas. Further analyses uncovered that application of 25-hydroxyvitamin D activated the Nrf2/CBR1 pathway, relieving the oxidative stress in BRL cells, suggesting that 25-hydroxyvitamin D regulates oxidative stress in offspring and induces the activation of the Nrf2/CBR1 pathway. Taken together, our study finds that maternal 25-hydroxyvitamin D deficiency is likely to result in offspring's MetS probably *via* abnormal nutrition transformation across placenta. Depression of the Nrf2/CBR1 pathway in both mothers and their offspring is one of the causes of oxidative stress leading to MetS. This study suggests that 25-hydroxyvitamin D treatment may relieve the offspring's MetS.

## Introduction

It has long been recognized that maternal nutrition along with environmental or physical conditions, has large impacts on the intrauterine environment, and ultimately, the fetal epigenome, including immune and metabolic functions in offspring (Sinclair et al., 2007; Chango and Pogribny, 2015). David Barker, an influential epidemiologist has proposed the “fetal programming hypothesis” (also known as “Barker hypothesis”), which states “the nourishment a baby receives from its mother, and its exposure to infection after birth, permanently ‘programme' the body's structure and metabolism, and determine its susceptibility to chronic disease in later life” (Barker et al., 1993; Cooper, 2013). Today, it is estimated that people with vitamin D deficiency account for 30% to 50% of the general population (Lee et al., 2008), and pregnant women are at particularly high risk of vitamin D deficiency (Özdemir et al., 2018). In recent years, increasing studies have shown that maternal vitamin D deficiency during pregnancy is associated with the occurrence of some metabolic syndrome (MetS) caused chronic diseases in the offspring, including obesity (Daraki et al., 2018), diabetes mellitus (Yu et al., 2018), insulin resistance (IR) (Zhang et al., 2014), cardiovascular diseases including hypertension (Weishaar and Simpson, 1987).

MetS is an assortment of cardiometabolic diseases and diabetic risk factors that has emerged as a global epidemic characterized by abnormal lipid metabolism, oxidative damage, IR and/or glucose tolerance (Samson and Garber, 2014). MetS is a main risk factor for heart and cerebrovascular diseases, as well as type 2 diabetes. Although the onset of symptoms in most cardiovascular diseases and type 2 diabetes occurs in adults or the elderly, there is strong evidence indicating childhood derived causes (Shalitin et al., 2016). To effectively control these chronic diseases, the prevention of MetS is extremely important. Vitamin D has been shown to function in the regulation of MetS. However, few studies have investigated the effects of maternal vitamin D deficiency during pregnancy on MetS in their offspring, especially adult offspring. Several studies have shown that Vitamin D deficiency during pregnancy could induce alterations in DNA methylation, and then result in changes in biological phenotype. Zhang et al. (2014) reported that maternal vitamin D deficiency during pregnancy resulted in IR in rat offspring, which was associated with inflammation and Iκbα methylation. A recent study found that Vitamin D deficiency during pregnancy affects the function of Th1/Th2 cells and methylation of IFN-γ gene in offspring rats (Jiao et al., 2019). However, compared to gene methylation, maternal oxidative stress has been indicated as the root origin promoting diverse metabolic disorders, and gestational oxidative stress has been determined to be the cause of their offspring adult metabolic diseases (Araujo et al., 2013). A study from Ito et al. (2016), as well as other recently published data, demonstrate the central role of oxidative stress in MetS development in fetal models. Therefore, protecting pregnant woman from oxidative stress may be a major therapeutic approach for the treatment of offspring MetS. Nrf2, as an important transcription factor regulating oxidative stress in cells and a central regulator of maintaining intracellular redox homeostasis, has aroused our widespread concern.

The nuclear factor erythroid 2 related factor 2 (Nrf2) is an important factor in cellular defense function that manages cell response to oxidative stress (Itoh et al., 1999). Recent studies have suggested that Nrf2 can function in different manners; it can control the expression of genes whose protein products are involved in the detoxification and elimination of reactive oxidants and electrophilic agents through conjugative reactions, thereby enhancing cellular antioxidant capacity; it also plays an important role in the regulation of glucose and lipid metabolism in obesity (Nguyen et al., 2009; Tanaka et al., 2008). A recent study by Xue et al. (2013) revealed that ablation of Nrf2 gene in the adipocytes of leptin deficient ob/ob mice (ob/ob mice) results in an even more severe obesity phenotype than typical ob/ob mice, which is coupled with aggravated IR, hyperglycemia, and hypertriglyceridemia. It is well known that the liver is extremely important in both lipid and carbohydrate metabolism, and Nrf2 is activated by oxidative stress to promote antioxidant defense in the liver. Nrf2 also regulates the hepatic metabolic homeostasis pathway *via* manipulation of target genes expression levels. A recent study from Wu et al. (2011) demonstrated that Nrf2 can induce the expression of genes involved in antioxidant defense, and promote generation of NADPH. Furthermore, the concentration of hepatic NADPH was reported to be low in the Nrf2 knockout mice and high in the hepatic specific Keap1 knockout mice. The activation of the Nrf2 signaling leads to an increase in the availability of NADPH to reduce the levels of ROS/RNS and therefore the lipid biosynthesis. Kitteringham et al. used 10-week-old wild type and Nrf2-KO mice fed with normal diet to assess the role of Nrf2 in liver lipid metabolism by proteomic analysis (Kitteringham et al., 2010). Studies by Shin et al. revealed that 1-[2-cyano-3,12-dioxooleana-1,9 (Samson and Garber, 2014)-dien-28-oyl]imidazole treatment decreased the mRNA expression of genes encoding the fatty acid synthesis enzymes including acetyl-CoA carboxylase 1 (ACC-1), acetyl-CoA carboxylase 2 (ACC-2), and fatty acid synthase in the liver of wild type mice fed with high-fat diet (60 kcal% fat) for 95 days (Shin et al., 2009), while Nrf2 deficiency increased lipogenesis and cellular ROS in mice with short-term high-fat diet or normal diet feeding. Carbonyl reductase 1 (CBR1) was reported to be able to protect cells against oxidative stress and cell death by inactivating cell membrane-derived lipid aldehydes (Kensler et al., 2007). Interestingly, in our previous research, we found that maternal 25-hydroxyvitamin D [25(OH)D] deficiency beginning around the time of conception can cause MetS in the adult offspring with high levels of oxidative stress and downregulated CBR1. Thus, the objective of this study was to investigate the potential roles of CBR1 in MetS and whether the promotion of CBR1 can protect adult offspring against MetS. Based on previous finding that Nrf2 directly regulates CBR1 transcription *via* the upstream ARE region (Miura et al., 2013), we propose that CBR1 might be a potential therapeutic target for reducing MetS in the adult offspring under the control of Nrf2. Hence, Nrf2/CBR1 signaling represents a potential therapeutic target for MetS *via* antioxidation.

In this paper, we hypothesize that maternal 25(OH)D deficiency or restriction during pregnancy has the potential to impact the metabolism of the offspring *via* suppressing the Nrf2/CBR1 pathway. The study aimed to investigate the effects and mechanism of maternal 25(OH)D restriction on metabolism in adult offspring.

## Materials and Methods

### Ethics Statement

All tissue samples were collected from animals described as follows. All protocols were approved by the Ethics Committee of the Laboratory Animal Centre, Wenzhou Medical University (no. wydw 2018-0031).

### Animals

Sprague-Dawley female and male rats (220 ± 20 g and 320 ± 20 g average body weight, respectively) were purchased from SLRC laboratory animal Co., Ltd (Shanghai, China). Animals were housed in a temperature-controlled room (constant temperature of 21 ± 2°C and a humidity of 60 ± 10%) with 12 h light/12 h dark rhythm cycle, and had free access to food and water. Female rats were randomly divided into two groups: a 25(OH)D deficiency group (VDD) which was given a 25(OH)D free diet (AIN-93G without 25(OH)D; AIN-93G, Keaoxieli Fodder, Beijing, China) and housed under incandescent light (free of ultraviolet radiation), and a control group (Con) which received a 25(OH)D-replete diet (AIN-93G, with 25(OH)D concentration = 1,000 IU/Kg) and normal light (with ultraviolet radiation) for 6 weeks. To encourage mating, the female rats were placed overnight with the male rats. Embryonic day 0 (E0) was defined as the day a sperm-positive vaginal smear was obtained. Pregnant rats (VDD-F0) was remain given a 25(OH)D free diet, and pregnant rats (Con-F0) received a 25(OH)D-replete diet throughout their pregnancy.

Pregnant rats were all individually housed. We defined age week 0 as the first day with the appearance of pups. Following birth, all male pups from both Con-F0 and early-VDD groups were immediately removed and suckled by dams in the control group and consequently placed under control light conditions. The male offspring were standardized to eight per litter. Once male offspring were weaned, all males were housed in individual cages and fed a 25(OH)D-replete diet, until 16 weeks of age.

To investigate any metabolic programming changes and expression levels of proteins of interest in the offspring, we chose four time points: 0 week (birth), 4 weeks (weaning), 8 weeks (near adulthood), and 16 weeks (adulthood). At each of these time points, ten offspring were selected from different litters (chosen randomly from different groups) and were weighed, fasted overnight, and anaesthetized. Blood was collected and then the pups were sacrificed by decapitation. The tissues were removed, weighed, snap-frozen in liquid nitrogen, and stored at −80°C. The placenta, liver, and pancreas sections that were designated for histology were rapidly formalin-fixed and paraffin embedded for immunohistochemistry analysis.

### Processing of Rat Liver, Placenta, and Pancreas Tissues

To detect ROS levels, tissue samples were pretreated before performing flow cytometry analysis. For pretreatment, adequate amounts of fresh tissues were cut up and 0.125% trypsin solution was added and incubated for 30 min at 37°C for tissue digestion. Tissue digestion was terminated with serum-containing medium. The cell suspension was transferred through a 200 mesh stainless steel screen, centrifuged at 402 (×g) for 5 min at 4°C. After centrifugation, ROS level was determined using flow cytometry.

To determine the protein concentrations in tissue samples, rat liver, placental, and pancreatic tissues were homogenized on ice using the radioimmunoprecipitation assay (RIPA, P0013B, Beyotime Biotechnology, China), followed by centrifugation at 16,200 (×g) for 15 min at 4°C. The supernatants were tested for the levels of superoxide dismutase (SOD) and protein levels were determined by Western blot analysis.

Tissues obtained for immunohistochemical (IHC) analysis were immediately fixed in 10% (v/v) neutral buffered formalin. Each specimen was then embedded in a paraffin block to prepare for IHC assay.

### Cell Culture

The rat liver cell line (BRL cell) was purchased from the Cell Bank of Shanghai. Cells were cultured in Dulbecco's modified Eagle's medium (DMEM; Gibco; Thermo Fisher Scientific, Inc., Waltham, MA, USA) containing 10% fetal bovine serum (FBS) (HyClone; GE Healthcare Life Sciences, Logan, UT, USA) in a humidified 5% CO_2_ incubator at 37°C. Only cells in the logarithmic phase from the third to eighth passage were used for experiments.

### RNA Interference of CBR1

*CBR1*-specific small interfering RNA (siRNA) [si*CBR1*-1 (si*C1*-1), si*CBR1*-2 (si*C1*-2), si*CBR1*-3 (si*C1*-3), GenePharma] were used to knock down *CBR1*. Cells were seeded in 6-well plates (2 × 10^5^ cells/well) and culture overnight in DMEM medium supplemented with 10% FBS without antibiotics. Cells were then transfected with either *CBR1* siRNA or a nonsilencing control siRNA using an siRNA reagent system (GenePharma) according to the manufacturer's protocol. Twenty-four hours after transfection, Western blot analysis was performed to detect the knock down efficiency of *CBR1*.

All the *CBR1*-specific siRNAs were effective at knocking down *CBR1* with the si*C1*-1 to be the most robust one. Following transfection, the cells were exposed to various treatments and analysis as described below.

### Cell Treatment

To identify the most effective concentration of 1,25-(OH)_2_D3 (740543, Sigma), we treated BRL cells with 1,25-(OH)_2_D3 at a series of concentrations (10 nM, 100 nM, or 1,000 nM) for 24 h, and then the Nrf2 protein levels were determined by Western blot analysis.

To determine the most effective concentration of Brusatol (Nrf2 inhibitor, CAS: 14907-98-3, Chengdu PureChem-Standard Co., Ltd, China), BRL cells were treated with different concentrations of Brusatol (20, 40, 80, or 160 nM) for 24 h, and then the Nrf2 protein levels were determined by Western blot analysis.

After initial experiments, cells were incubated under different conditions: 1,25-(OH)_2_D3 (1,000 nM), 1,25-(OH)_2_D3 (1,000 nM) + Brusatol (160 nM), or 1,25-(OH)_2_D3 (1,000 nM) + si*C1*-1. Nrf2 and CBR1 expression levels were measured by Western blot and real-time PCR.

### CHIP Assay

BRL cells treated with or without VitD3 for 48 h were used for the cross-linking with formaldehyde. For the immunoprecipitation, normal rabbit IgG and anti-Nrf2 antibody (CST, 12721S) were added respectively and incubated at 4℃ for 4 h. After DNA extraction, the CBR1 promoter region was amplified by PCR using the following primers:

Forward: 5′-GTTCTTCACTGCCACGTG-3′

Reverse: 5′-TGAACCATCCCTACCCCTC-3′.

### Determination of the Levels of TG, HDL-C, FBG and INS

The serum levels of triglycerides (TG), high-density lipoprotein (HDL-C), fasting blood glucose (FBG) and insulin (INS) were assayed using the appropriate assay kits. The FBG biochemical kit was purchased from Applygen (E1010, China). The INS, TG, and HDL-C ELISA kits were purchased from Bioswamp (RA20092, RA20187, RA20667, USA).

The serum samples were harvested and used for examining related indexes according to the manufacturer's protocols. Serum chemistry levels were measured using an automated analyzer (1681130-4B, Bio-Rad, America).

Homeostasis model assessment (HOMA) models were used to estimate IR (HOMA-IR), insulin sensitivity (HOMA-S), and β-cell function (HOMA-β), which were calculated by the following equations: HOMA-IR = [fasting insulin (mU/L) × fasting glucose (mmol/L)]/22.5, HOMA-S = 22.5/[fasting insulin (mU/L) × fasting glucose (mmol/L)], and HOMA-β= [20 × fasting insulin (mU/L)]/[fasting glucose (mmol/L) − 3.5].

### ROS Detection by Flow Cytometry

To confirm the levels of ROS in BRL cells after treatments with Brusatol, VD3, or si*C1*-1, all cells were digested with 0.25% trypsin, and 500 μL of 1 μM CM-H2DCFDA (C6827, Invitrogen) were added to each tube and incubated at room temperature in the dark for 30 min. Next, cells were washed twice with phosphate-buffered solution (PBS) and gently resuspended in DMEM medium containing 10% FBS, and then analyzed using flow cytometry (ACEA NovoCyte, China). For the tissues, single cell suspension prepared from placenta, liver, and pancreas tissues of rats.

### Detection of the SOD Levels

The harvested protein supernatants were used to evaluate the levels of SOD in tissues by following the SOD detection kit protocol provided by the manufacturer (A001-1-1, Jiancheng, China).

### Hematoxylin and Eosin (H&E) Staining

Maternal placentas were immersed in 4% paraformaldehyde and incubated for 4 h, and then transferred to70% ethanol. Individual lobes of placenta biopsy material were placed in processing cassettes and dehydrated through a serial alcohol gradient, and embedded in paraffin wax blocks. Before staining, 5-µm thick placenta tissue sections were dewaxed in xylene, and rehydrated through decreasing concentrations of ethanol, and washed in PBS. The sections were then stained with H&E.

### IHC Staining

Nrf2 (ab186825, Abcam), CBR1 (ab89433, Abcam), IL-1β (ab9722, Abcam), IL-6 (21865-1-AP, Proteintech), and TNFα (BA0131, Boster) were used for the IHC detection of protein expression in the trimethylamine (TMA). Nrf2 and CBR1 antibodies were both used at a 1:200 dilution. Endogenous peroxidase was inhibited by incubation with freshly prepared 3% hydrogen peroxide with 0.1% sodium azide. Non-specific staining was blocked with 0.5% casein and 5% normal serum. The TMA samples were incubated with biotinylated antibodies and horseradish peroxidase. Staining was developed with diaminobenzidine substrate and the sections were counterstained with hematoxylin. Negative controls were obtained by using PBS instead of Nrf2 or CBR1 antibody. The expressions of Nrf2 or CBR1 were quantified as previously described (Zhang et al., 2016).

### Western Blot

The protein concentrations of the samples were measured using a BCA protein assay kit (Beyotime Biotechnology, China). The samples containing equal amounts of proteins (30 μg) were denatured in sample loading buffer and separated by sodium dodecyl sulfate–polyacrylamide gel electrophoresis, followed by a transfer to a nitrocellulose membrane (Hybond-ECL, Amersham Biosciences). Then membranes were blocked with 5% (w/v) non-fat dried milk in a Tris-buffered saline with Tween 20 (TBST), and incubated with primary antibodies (anti-Nrf2 (1:500 dilution in TBST; Rabbit, OmnimAbs Co., H-300) and anti-CBR1 (1:500 dilution in TBST; rabbit, Affinity Co., DF7346)) overnight at 4°C. Then membranes were washed three times and incubated with horseradish peroxidase-conjugated anti-goat (for Nrf2) or anti-rabbit (for CBR1) second antibodies (ab6789, Abcam, USA) for 2 h. Immunoreactive bands were visualized using ECL^®^ chemiluminescence reagents (Amersham Biosciences)according to the manufacturer's instructions, followed by exposure to X-ray ﬁlms (Sigma Z370398). Films were analyzed with Quantity One Software (BioRad Laboratories), and the resulting absorbance values were expressed as the percentage variation of the control group values. The expression of glyceraldehyde 3-phosphate dehydrogenase (GAPDH) was used as loading control.

### RNA Extraction and Quantitative Real-Time PCR (qRT-PCR)

Total RNA was extracted from various rat tissues using RNA extraction kit (EZB-RN001, Ezbioscience Co., USA) according to the manufacturer's instructions, and used for reverse transcription into cDNA by applying RevertAid First Strand cDNA synthesis Kit (#K1622, Thermo Co., USA). The cDNA was then subjected to RT-qPCR analysis using SsoAdvance Universal SYBR Green Supermix (cat#172-5274, Bio-Rad Co., USA), and gene expression was quantified as previously described (Magalhaes et al., 2013). The *Nrf2* and *CBR1* amplification signals were normalized to *GAPDH* expression. Gene-specific primer sequences used for qRT-PCR are listed as follow:

Rat *CBR1* Forward: 5′-TTGACGAGACCTACCTGATGTT-3′;

Rat *CBR1* Reverse: 5′-TGTGGATGATGATGCTCTTCTG-3′.

Rat *Nrf2* Forward: 5′-GAGACGGCCATGACTGAT-3′;

Rat *Nrf2* Reverse: 5′-TGAGGGGATCGATGAGTA-3′.

Rat *GAPDH* Forward: 5′-GGTGGACCTCATGGCCTACA-3′;

Rat GAPDH Reverse: 5′- CTCTCTTGCTCTCAGTATCCTTGCT-3′.

### Statistical Analysis

Results are expressed as means ± SD. The unpaired 2-tailed Student's *t*-test for two experimental groups and two-way ANOVA for multiple groups were employed to assess statistical differences. A value of *P* < 0.05 denotes statistical signiﬁcance, *P* < 0.01 denotes a higher level of signiﬁcance and *P* < 0.001 denotes a very high level of statistical signiﬁcance. Statistical analyses were done using GraphPad Prism 6 (GraphPad Software, Inc.).

## Results

### Maternal 25(OH)D Deficiency Enhances Oxidative Stress, Leading to MetS in Mothers and Their Offspring

To determine the validity of the model, the maternal vitamin D level was measured on embryonic day 0 (E0) and embryonic day 21 (E21). As shown in **Supplementary Figure 1**, compared with the control group, the mean serum 25(OH)D concentrations of vitamin D deficiency group were markedly reduced in the dams on E0 and E21, while there was no significant difference in body weight or adjusted energy intake between the two groups, indicative of success of the model (**Supplementary Figures 2A, B**).

**Figure 1:**
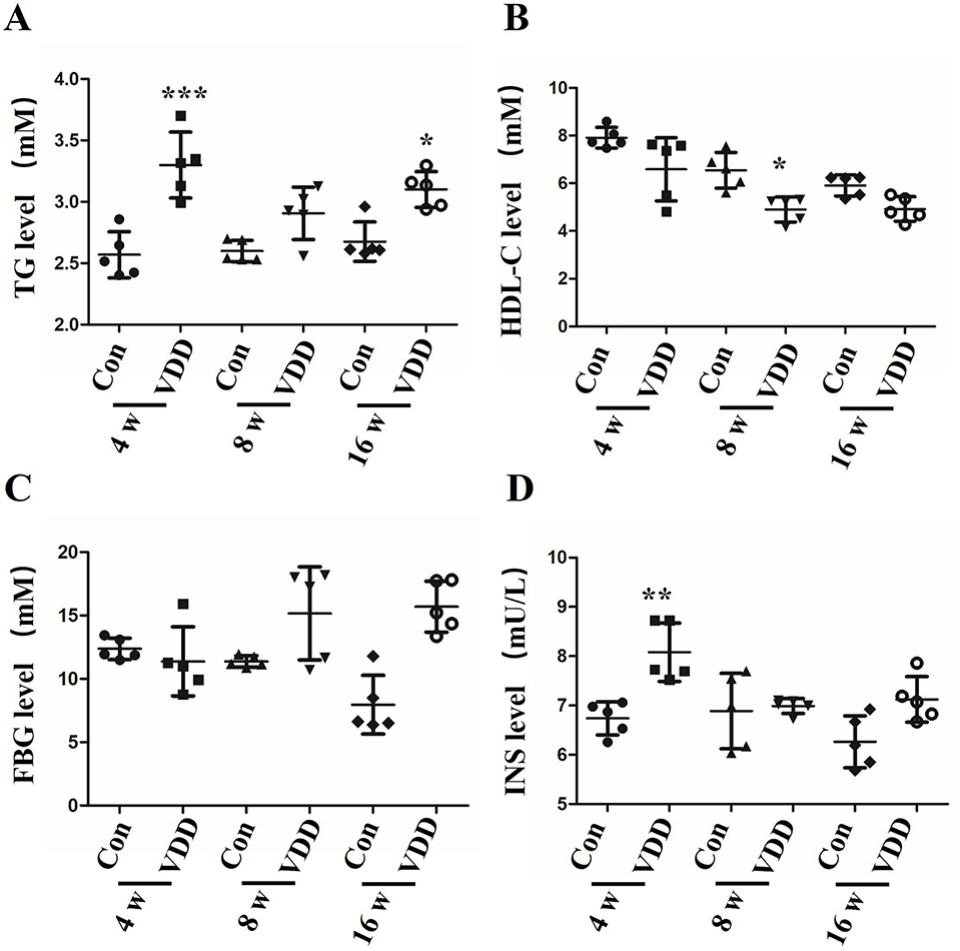
The influence of maternal 25-hydroxyvitamin D deficiency on offspring metabolic function. **(A)** The blood triglycerides (TG), **(B)** the high-density lipoprotein cholesterol (HDL-C), **(C)** the fasting blood glucose (FBG), and **(D)** the fasting insulin (INS) were monitored by ELISA assay in offspring blood at week 4, 8, and 16. The data are expressed as the means ± SD of at least three independent experiments. n = 5 rats per group. *P < 0.05, **P < 0.01, ***P < 0.001 represents the comparisons between maternal 25-hydroxyvitamin D deficiency group (VDD) and normal control group (Con). Normal group, Con; maternal 25-hydroxyvitamin D deficiency group, VDD; week, w.

It has been reported that MetS in rats caused by maternal 25(OH)D deficiency may be mediated through several different mechanisms, including increased oxidative stress and IR (Zhang et al., 2014). To explore the impact of 25(OH)D deficiency on MetS in maternal and offspring rats *in vivo*, we chose to examine several metabolic indicators, including blood TG levels, HDL-C levels, fasting insulin (INS) levels, and the FBG levels of pups from 25(OH)D deprived pregnant rats. We found that the levels of TG, FDG, and INS were significantly increased in the 25(OH)D deprived pups (**Figures 1A, C, D**), while HDL-C levels were significantly decreased (**Figure 1B**) compared with control group (**Table 1**).

**Table 1:** Fasting variables of the control and VDD group of offspring at different time points.

Variable	4w		8w		16w	
	Control (n = 5)	VDD (n = 5)	Control (n = 5)	VDD (n = 5)	Control (n = 5)	VDD (n = 5)
TG (mmol/l)	2.57 ± 0.19	3.30 ± 0.27***	2.60 ± 0.09	2.91 ± 0.21	2.68 ± 0.16	3.10 ± 0.14*
HDL-C (mmol/l)	7.91 ± 0.43	6.59 ± 1.33	6.54 ± 0.75	4.9 ± 0.52*	5.9 ± 0.44	4.92 ± 0.51
INS (mU/L)	6.74 ± 0.34	8.08 ± 0.59**	6.89 ± 0.76	6.99 ± 0.15	6.26 ± 0.53	7.12 ± 0.46
FBG (mmol/l)	12.37 ± 0.85	11.38 ± 2.72	11.38 ± 0.45	15.16 ± 3.67	7.96 ± 2.31	15.69 ± 2.01***
HOMA-IR	3.7 ± 0.23	4.11 ± 1.18	3.49 ± 0.46	4.72 ± 1.18	2.20 ± 0.55	4.98 ± 0.79***
HOMA-S	0.27 ± 0.02	0.26 ± 0.06	0.29 ± 0.04	0.22 ± 0.06	0.48 ± 0.11	0.20 ± 0.03***
HOMA-β	15.33 ± 1.91	22.19 ± 6.98	17.48 ± 1.72	13.15 ± 4.69	33.42 ± 13.43	11.92 ± 1.99***
calcium (mmol/l)	2.53 ± 0.16	2.54 ± 0.18	2.41 ± 0.17	2.45 ± 0.21	2.47 ± 0.13	2.44 ± 0.16

FBG, fasting blood glucose; HOMA-IR, homeostasis model assessment of insulin resistance; HOMA-S, insulin sensitivity; HOMA-β, β-cell function; TG, triglyceride; HDL-C, high-density lipoprotein cholesterol; n. Normally distributed data are presented as means ± SD; *P < 0.05, **P < 0.01, ***P < 0.001 vs. control group.

**Figure 2:**
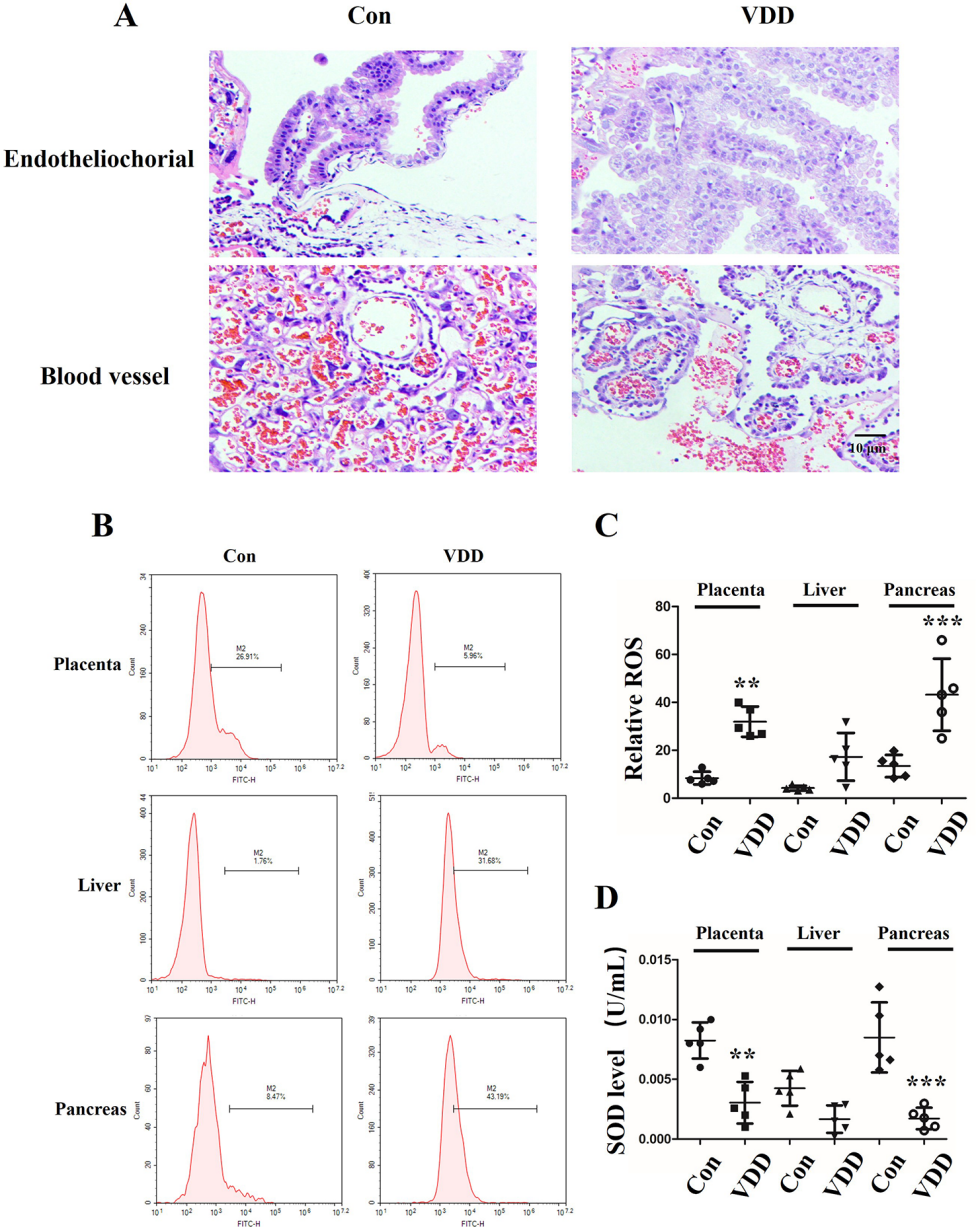
Structure change of the placenta and ROS accumulation under maternal vitamin D deficiency status. **(A)** HE staining for blood vessel numbers and amniotic epithelium information in SD rat placenta on gestation day 21. **(B)** The ROS levels in maternal placenta, liver, and pancreas were assessed by flow cytometry using the CM-H2DCFDA fluorescence probe. **(C)** The percentage of fluorescence intensity was calculated as relative ROS level in maternal placenta, liver, and pancreas. **(D)** The SOD levels were detected in the maternal in maternal placenta, liver, and pancreas. The data are expressed as the means ± SD of at least three independent experiments. n = 5 rats per group. **P < 0.01, ***P < 0.001 represents the comparisons between maternal 25-hydroxyvitamin D deficiency group (VDD) and normal control group (Con). Normal group, Con; maternal 25-hydroxyvitamin D deficiency group, VDD; week, w.

**Figure 3:**
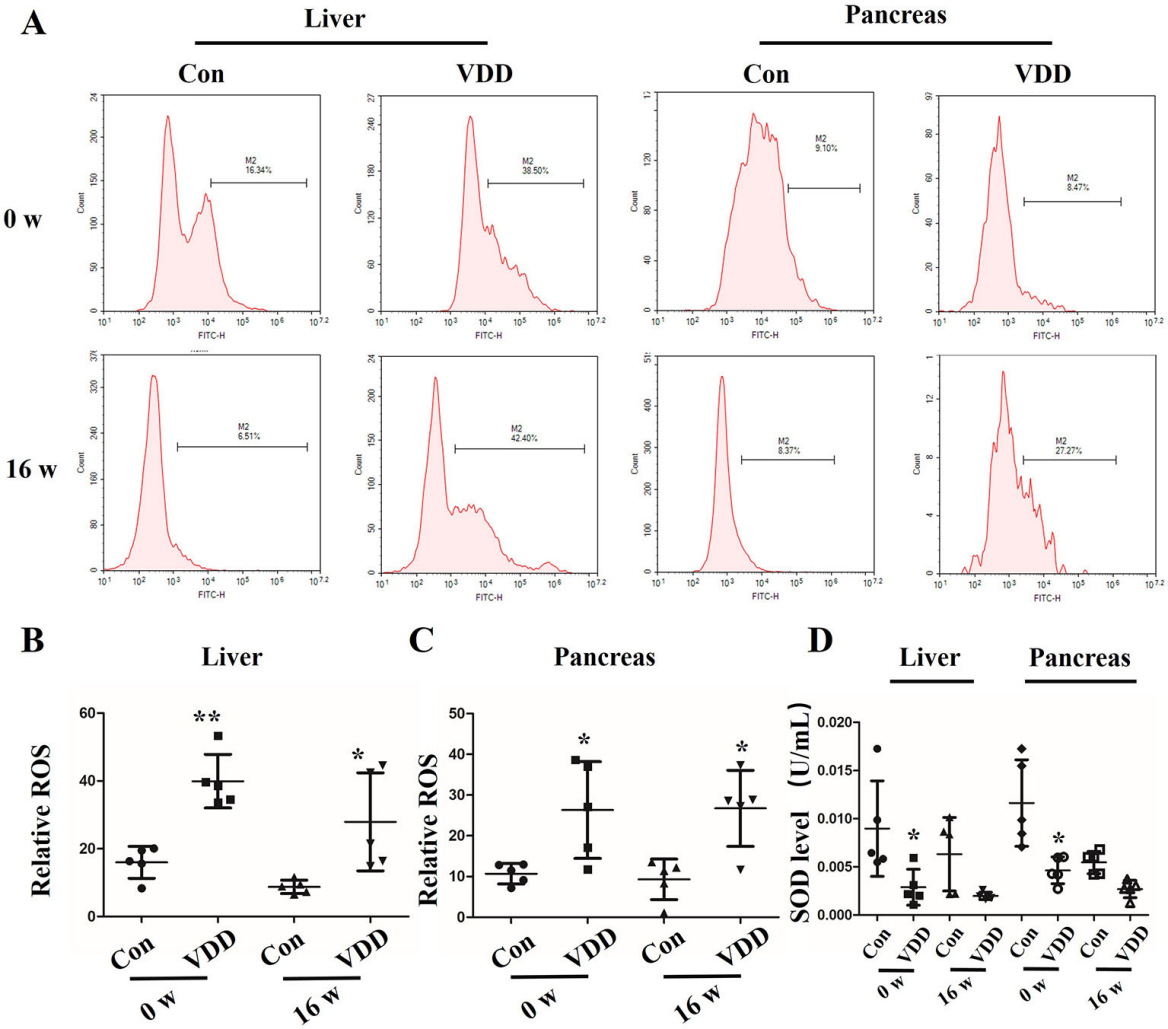
Maternal 25-hydroxyvitamin D deficiency decreases the antioxidation ability in offspring liver and pancreas tissues. **(A)** The liver and pancreas ROS levels in offspring at week 0 or 16 was assessed by flow cytometry using the CM-H2DCFDA fluorescence probe. **(B, C)** The percentage of fluorescence intensity was calculated as relative ROS level in liver **(B)** and pancreas **(C)** at week 0 and 16. **(D)** SOD levels were detected in the offspring liver and pancreas at week 0 and 16. Mean ± SD, n = 5 rats per group. *P < 0.05, **P < 0.01, vs. control group. Normal group, Con; maternal 25-hydroxyvitamin D deficiency group, VDD; week, w.

We also observed structure change of the placenta and ROS accumulation under maternal vitamin D deficiency status. Interestingly, compared with control group, the blood vessel number in vitamin D deficiency group was increased (2.40 ± 1.17 vs. 6.60 ± 1.71, *P* < 0.001) and amniotic epithelium had more branches and a larger amount (**Figure 2A**). Meanwhile, the level of SOD was decreased in maternal tissues (**Figures 2B–D**). Moreover, increased ROS accumulation was also observed in the 25(OH)D deficiency pup offspring, which was contrary to the changes in SOD levels. Overall, as pups got older, the differences induced by 25(OH)D deficiency became increasingly significant (**Figure 3**).

### Maternal 25(OH)D Inhibits Activation of the Nrf2/CBR1 Pathway in Maternal Placenta, Liver, and Pancreas and Offspring’s Liver and Pancreas

**Figure 4:**
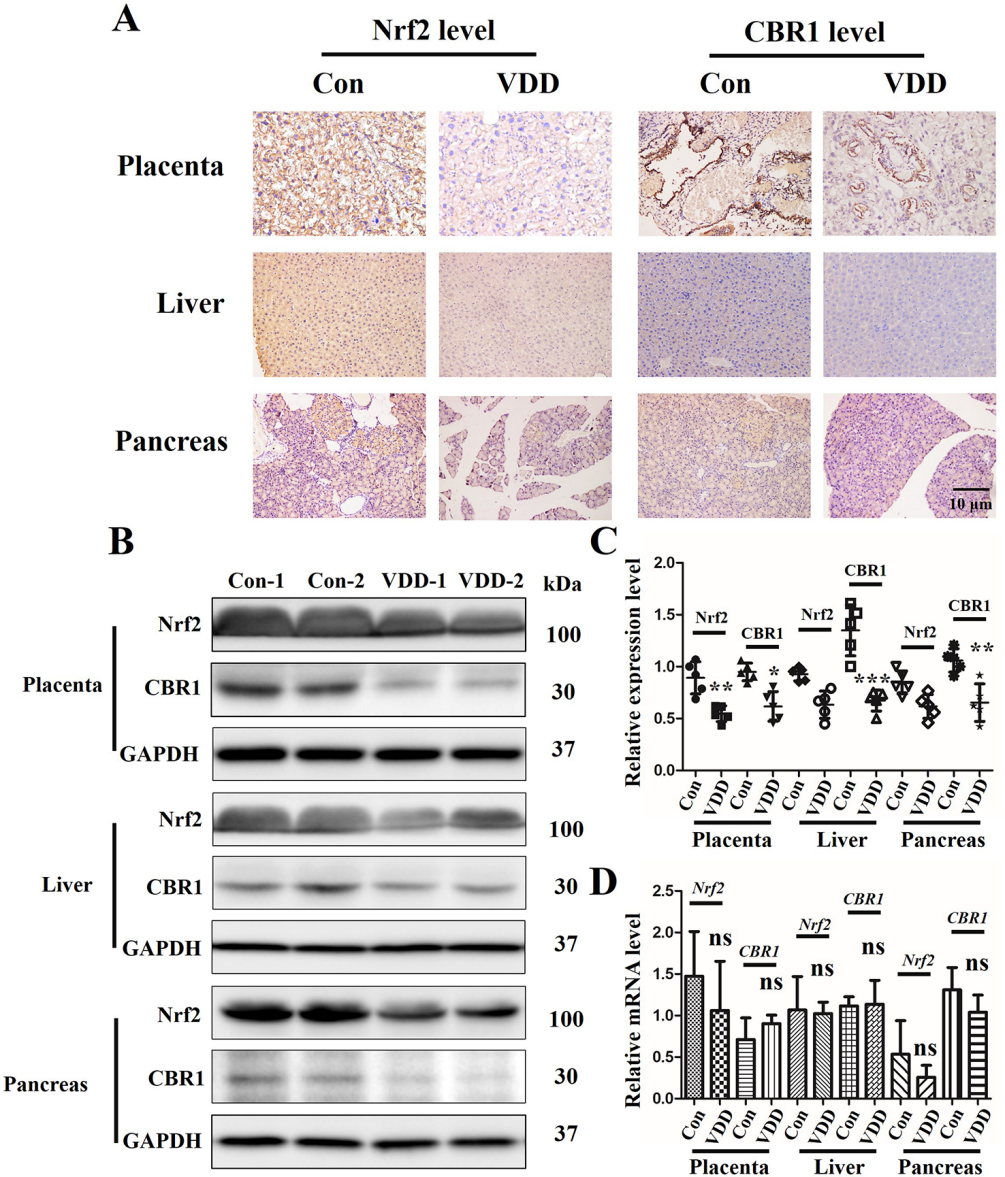
The effect of Maternal 25-hydroxyvitamin D deficiency on the expression level of Nrf2 and CBR1 in maternal placenta, liver, and pancreas tissues. **(A)** Nrf2 and CBR1 immunohistochemical staining in maternal placenta, liver, and pancreas tissues obtained in normal group and 25-hydroxyvitamin D deficiency group (magnification, ×200). **(B**–**D)** Application of Western blot **(B**, **C)** and qT-PCR **(D)** to ascertain the protein and mRNA expression levels, respectively, in maternal placenta, liver, and pancreas tissues. Mean ± SD, n = 5 rat per group. *P < 0.05, **P < 0.01, **P < 0.001 vs. control group; ns, no significance. Normal group, Con, Con-1, and Con-2 represent different rats; maternal 25-hydroxyvitamin D deficiency group, VDD, VDD-1, and VDD-2 represent different rats.

As Nrf2 plays a critical role in the cellular response to oxidative stress (Tanaka et al., 2008), we speculated that 25(OH)D deficiency may downregulate Nrf2 and its downstream target gene *CBR1*, which leads to the development of MetS. IHC analysis showed that the establishment of the 25(OH)D deficiency rat model induced reduction in Nrf2 and CBR1 protein expression in the maternal placenta, liver, and pancreas (**Figure 4A**), which was further confirmed by Western blot analysis (**Figures 4B, C**). However, we did not detect a significant reduction in the mRNA levels of Nrf2 and CBR1 in the maternal placenta, liver, and pancreas in the maternal 25(OH)D deficiency cohort (**Figure 4D**).

**Figure 5:**
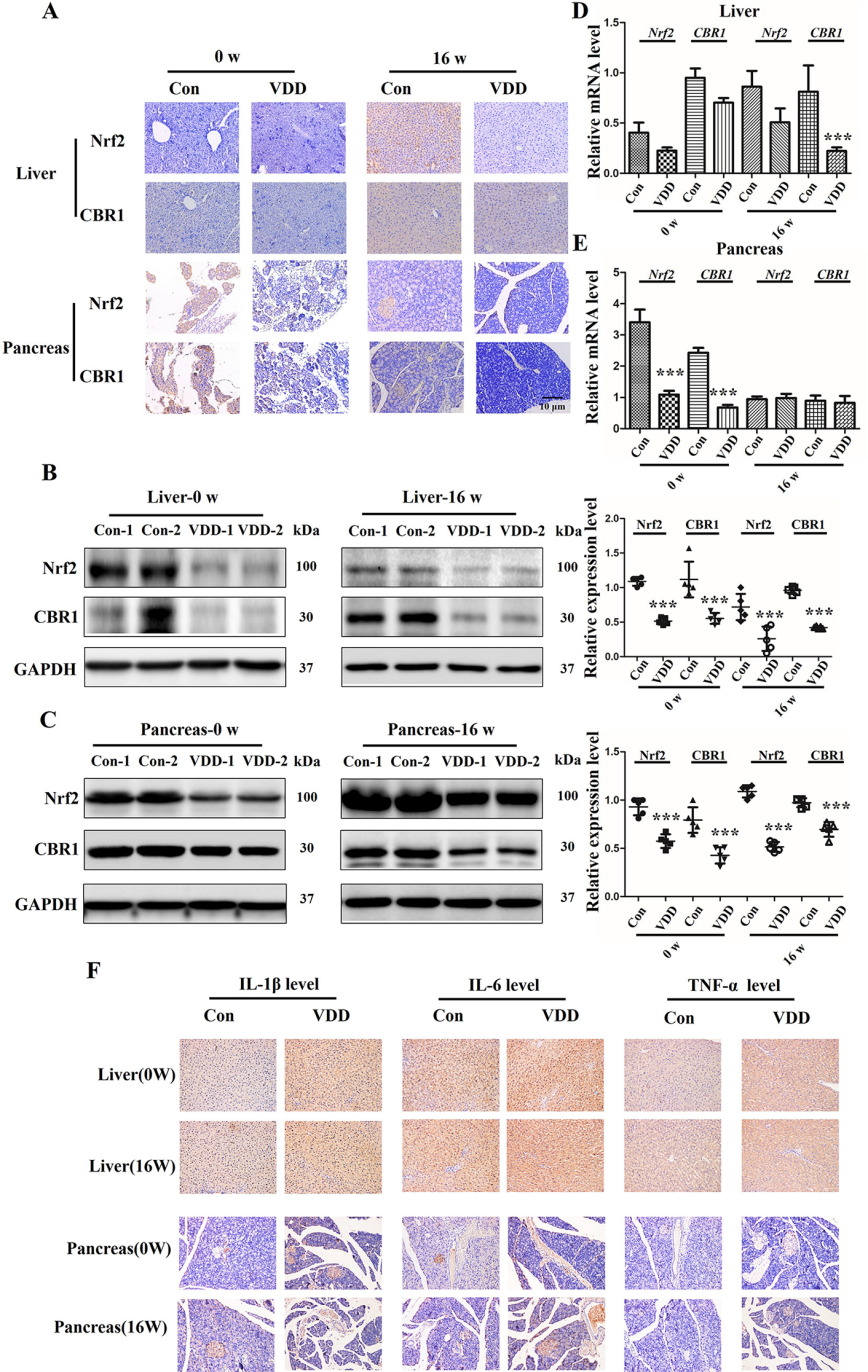
Maternal 25-hydroxyvitamin D deficiency altered expression levels of Nrf2, CBR1, and inflammatory cytokine in offspring at week 0 and 16. **(A)** Nrf2 and CBR1 immunohistochemical staining in offspring liver and pancreas tissues obtained in normal group (Con) and 25-hydroxyvitamin D deficiency group (VDD) at week 0 and 16 (magnification, ×200). **(B**, **C)** Western blotting analysis was used to detect theNrf2 and CBR1 expression level at week 0 and 16 in offspring rat liver **(B)** and pancreas **(C)**. Densitometry data for Nrf2 and CBR1 from the blots shown were normalized for analysis to GAPDH, the loading control. **(D, E)** The Nrf2 and CBR1 levels in offspring liver **(D)** and pancreas **(E)** at week 0 and 16 were determined by qRT-PCR. **(F)** IL-1β, IL-6and TNF-α IH staining in offspring liver and pancreas tissues obtained in normal group (Con) and 25-hydroxyvitamin D deficiency group (VDD) at week 0 and 16 (magnification, ×200). Mean ± SD, n = 5 rats per group. ***P < 0.01, vs. control group. Normal group, Con, Con-1, and Con-2 represent different rats; maternal 25-hydroxyvitamin D deficiency group, VDD, VDD-1, and VDD-2 represent different rats; week, w.

Analyses of offspring liver and pancreas tissues at week 0 and 16 time points were performed using IHC and Western blot and the results revealed an interesting phenomenon. We observed that the Nrf2 and CBR1 protein levels were all significantly decreased vitamin D deficiency group compared with control group (**Figures 5A–C**). As for the mRNA levels of *Nrf2* and *CBR1*, we found different expression levels in different tissues at different time points. In liver tissue, we found both *Nrf2* and *CBR1* mRNA levels were more significantly decreased in the 25(OH)D deficiency rat offspring at week 0 and week 16 (**Figure 5D**). In pancreas tissue, we found significantly reduced mRNA levels of *Nrf2* and *CBR1* at 0 week, while this decrease was rescued to wild-type level at week 16 (**Figure 5E**). Meanwhile, three important genes (HO-1, NQO1, and GCLC) regulated by Nrf2 were examined. qRT-PCR results showed that the mRNA levels of HO-1, NQO1, and GCLC were downregulated in the 25(OH)D deficiency rat offspring at week 0 and week 16, which were approximately consistent with the change trend of Nrf2 (**Supplementary Figure 3**). Because inflammation usually accompanies oxidative stress (Mangge et al., 2014), we also measured the inflammation markers in tissues. Results shown that, the levels of IL-1β, IL-6, and TNF-α were significantly higher in the vitamin D deficiency group than in the control group at week 0 and 16 (**Figure 5F**, **Supplementary Figure 4**).

### 25(OH)D Activates the Nrf2/CBR1 Pathway to Relieve the Oxidative Stress in BRL Cells

Considering the known anti-oxidation roles of the Nrf2/CBR1 pathway, a representative rat liver BRL cell line was employed to further investigate the metabolic function (Zhong et al., 2017). Results showed that the expression levels of Nrf2 and CBR1 were both efficiently reduced *via* application with Brusatol and si*CBR1*. Brusatol is a specific inhibitor of Nrf2, and to determine the optimum concentration needed for successful knockdown, BRL cells were treated with different concentrations of Brusatol, and we confirmed the optimum concentration of Brusatol to be 160 nM (**Supplementary Figure 5A**). To determine the best siRNA for CBR1 knockdown, three different *CBR1* speciﬁc siRNAs were independently transformed into BRL cells. All three siRNAs produced a dramatic reduction in CBR1 levels in comparison to control cells (**Supplementary Figure 5B**). On this basis, si*CRB1*-1 was selected for the following experiments.

**Figure 6:**
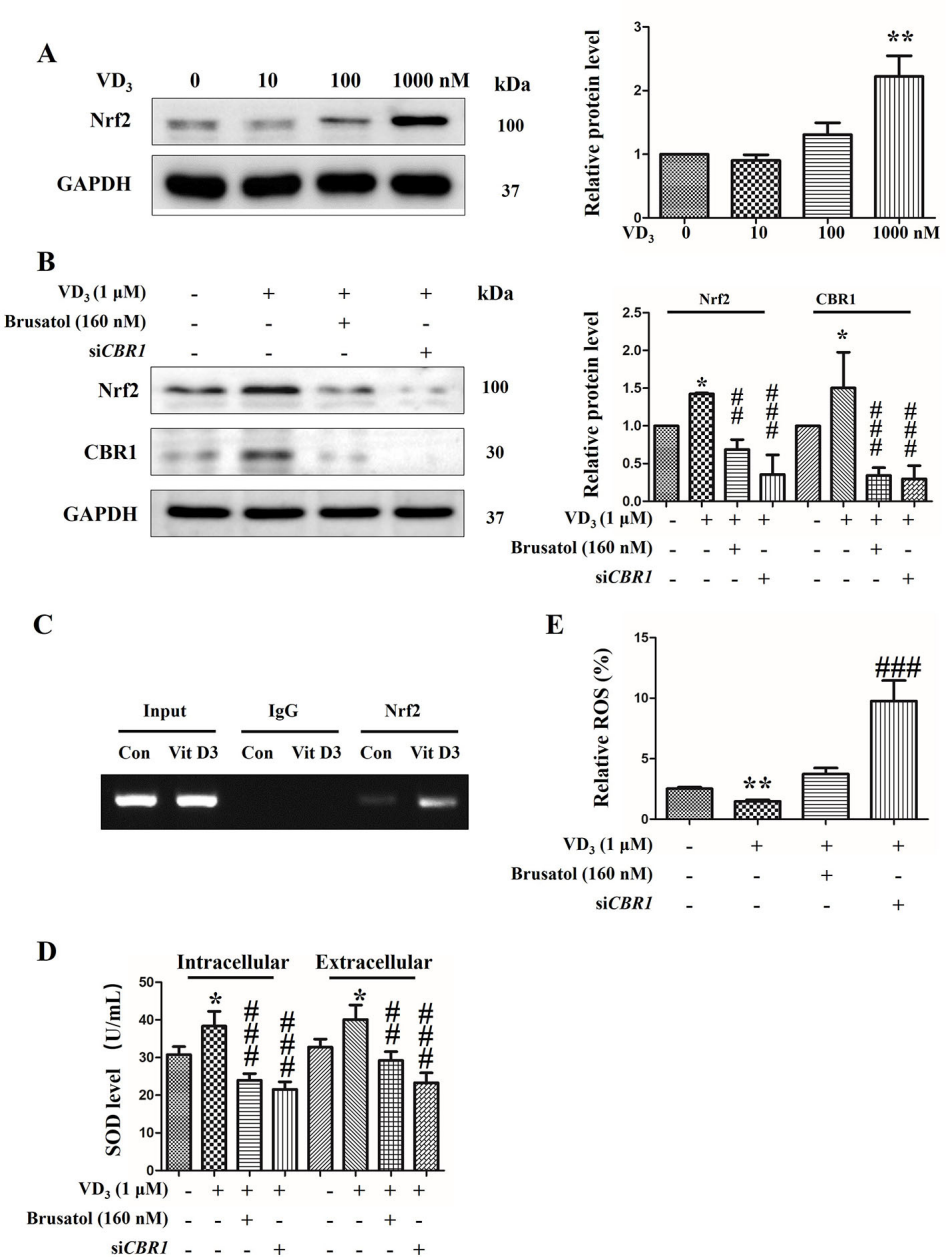
25-Hydroxyvitamin D activated Nrf2/CBR1 pathway and upregulated the antioxidative activity of BRL cells. **(A)** BRL cells were exposed to 25-hydroxyvitaminD (VD3) at 0, 10, 100, 1,000 nM for 24 h, and thenNrf2 levels were evaluated by Western blot. **(B)** BRL cells were exposed to VD3 (1 μM) with or without Brusatol (160 nM) or siCBR1. Next, the level of Nrf2 was determined by Western blotting. **(C)** CHIP assay was performed using BRL cells treated with1 µM VitD3)/without VitD3 (Con) for 24 h. Normal rabbit IgG and Anti-Nrf2 antibody were used for immunoprecipitation and the CBR1 promoter region was amplified by PCR. **(D, E)** BRL cells were treated with VD3 (1 μM) with or without Brusatol (160 nM) or siCBR1. Then, the level of SOD was calculated by SOD assay kit **(C)** and the ROS accumulation was determined *via* flow cytometry using the CM-H2DCFDA fluorescence probe **(D)**. The results are expressed as mean ± SD of three independent determinations. *P < 0.05, **P < 0.01, vs. control group, untreated group, ^##^ *P* < 0.01, ^###^ *P* < 0.001, vs. VD3 (1 μM) treated group.

Next, to determine the effect of 25(OH)D on the Nrf2/CBR1 pathway, we treated BRL cells with 25(OH)D and examined the Nrf2 expression levels. BRL cells treated with 25(OH)D demonstrated a significant upregulation of Nrf2 expression levels, and this effect was found to be 25(OH)D dose dependent (**Figure 6A**). Next, BRL cells were treated with 25(OH)D and Brusatol or si*CBR1*. We found that both Brusatol and si*CBR1* completely blocked the 25(OH)D induced upregulation of Nrf2 and CBR1 expression (**Figure 6B**). Meanwhile, the CHIP assay indicted that the binding ability of Nrf2 to the CBR1 promoter region was greatly enhanced after VitD3 treatment (**Figure 6C**). These results suggest that 25(OH)D can promote CRB1 transcription by activating the transcription factor Nrf2.

We next explore how the activation of the Nrf2/CBR1 pathway is induced by 25(OH)D and oxidative stress. Treatment of BRL cells with 25(OH)D caused a significant increase in SOD production. This increase was inhibited by co-treatment with Nrf2 inhibitor Brusatol, or si*CBR1*, which reduces CBR1 expression levels (**Figure 6D**). While the production of ROS was decreased upon addition of 25(OH)D, this was reversed by co-addition with Brusatol or si*CBR1* (**Figure 6E**). These results demonstrate that 25(OH)D can ameliorate oxidative stress *via* activating the Nrf2/CBR1 pathway.

## Discussion

**Figure 7:**
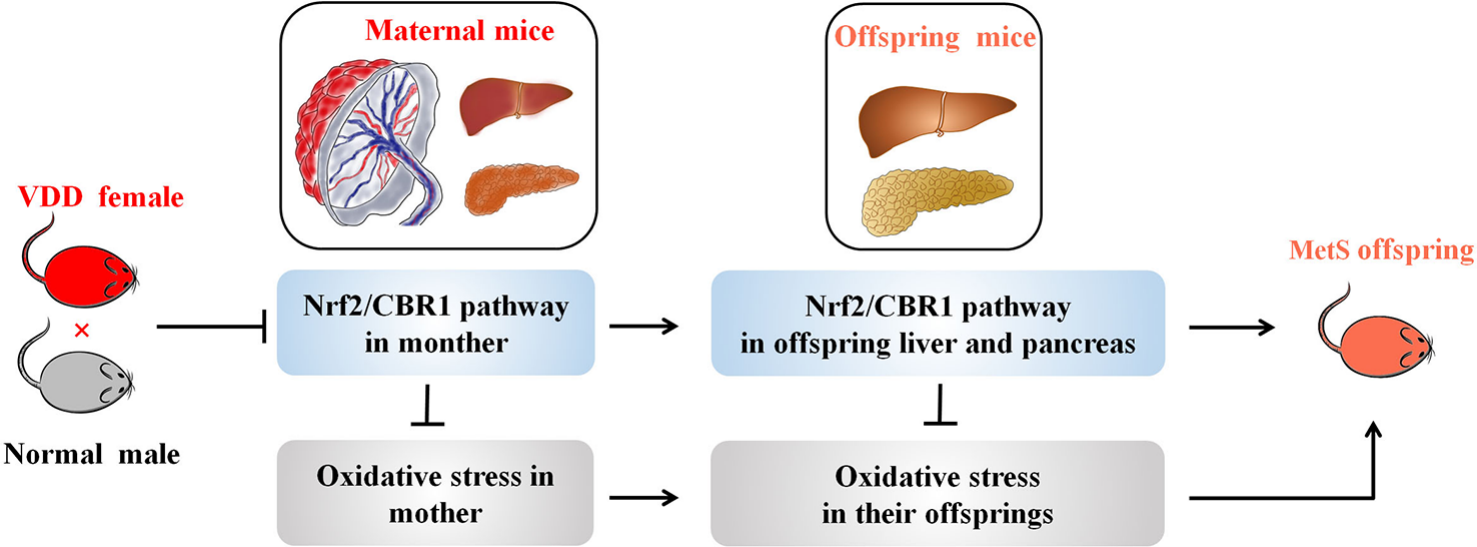
A working model illustrating the effect of maternal VDD on MetS in their offspring. A working model depicting how maternal VDD effects offspring metabolic function was shown. Maternal VDD leads to the inhibition of Nrf2/CBR1 pathway both in mother and their offspring. Further, the depression of the Nrf2/CBR1 pathway in both the mother and her offspring is the main cause of oxidative stress leading to MetS.

Here, we reveal evidence that maternal 25(OH)D deficiency beginning around the time of conception can cause MetS in the adult offspring, and that this may be induced by higher levels of oxidative stress in the offspring. We observed increased levels of TG, FBG, INS, and HOMA-IR, and decreased level of HDL-C in peripheral blood of the offspring from mothers that experienced 25(OH)D deficiency during pregnancy (**Figure 1**), indicating that maternal 25(OH)D deficiency induced MetS in their offspring, which is consistent with the findings from Huaqi Zhang et al. (Zhong et al., 2017). In addition, we found 25(OH)D deficiency increased ROS levels and decreased SOD levels. In both the liver and pancreas, both Nrf2 and CBR1 levels were downregulated in the offspring (**Figures 3** and **5**) suggesting that the oxidative stress induced by maternal 25(OH)D deficiency in offspring is correlated with the expression of Nrf2 and CREB1. Also there is convincing evidence that high level of inflammation is a major risk factor for the development of IR. By detecting liver inflammation level, we found that IL-1β, IL-6and TNF-α were significantly elevated in livers and pancreas of the offspring in the vitamin D deficiency group compared to control group at all time points (**Figure 5**). We also found that vitamin D deficiency can induce changes in the structure of maternal's placenta. Compared with the control group, the blood vessel and amniotic epithelium amounts and the amniotic epithelium branches in vitamin D deficiency group were significantly increased. In summary, our results suggest oxidative stress and inflammation precede MetS in the pups and change the structure of the maternal's placenta under 25(OH)D deficiency (**Figure 7**).

To explore the role of oxidative stress in promotion of MetS, we examined the Nrf2/CBR1 pathway, which is known to play a role in anti-oxidative stress processes. Takeshi Miura et al. identified that Nrf2, a novel transcriptional regulator, regulates the transcription of antioxidant enzymes, and CBR1, an antioxidant enzyme, is regulated by Nrf2 (Miura et al., 2013). Previous studies found that transcriptional upregulation of CBR1 is controlled by Nrf2, and intracellular ROS levels and lipid peroxidation are increased in cells transfected with CBR1 siRNA, as well as in cells treated with the selective CBR1 inhibitor, Hydroxy-PP-Me in C2C12 mouse myoblasts (Wu et al., 2011). Here we found that Nrf2 and CBR1 levels were decreased, ROS levels were increased, and SOD levels were decreased in the maternal placenta, hepatic, and pancreas of 25(OH)D deficient pregnant rats. This suggests that oxidative stress may begin with maternal 25(OH)D deficiency during pregnancy, and this effect may be systemic and not limited to certain organs (**Figures 2** and **4**). Moreover, repression of Nrf2 and CRB1 expression through use of a specific inhibitor or siRNA was also found to promote the production of ROS and eliminate the production of SOD, even when BRL cells were co-treated with 25(OH)D (**Figure 6**). Taken together, these results demonstrate that the Nrf2/CBR1 pathway plays an essential role in both the course of oxidation stress induced by maternal 25(OH)D deficiency in the offspring and the 25(OH)D deficient mother. Further research is needed to understand whether Nrf2 regulates the transcription of other antioxidant genes other than *CBR1*.

In light of Nrf2 and CBR1 expression reduction in 25(OH)D deficient maternal placenta (**Figure 4**), we explored the role of the placenta in the process of maternal 25(OH)D deficiency induced MetS in offspring. The placenta is a multifunctional organ enabling optimal fetal growth (Chatuphonprasert et al., 2018). The fetus develops in the womb and depends on the placenta for receiving nutrients from the mother. The proper transfer of nutrition from the maternal circulation across the placenta to the fetal circulation is essential for metabolism homeostasis and the control of fetal development (Arabin and Baschat, 2017). Barker and his team confirmed that in case of failure of adaptation or inadequate placental development, fetal survival, or growth and development are severely endangered, and developmental programming of adult diseases may occur (Barker and Thornburg, 2013; Burton et al., 2016). Earlier studies had verified that the down-regulation of peroxisome proliferator activated receptor gamma in placentas may predict hyperglycemia in offspring at young adulthood (Zhao et al., 2018). Analogously, offspring MetS induced by oxidative stress resulting from maternal 25(OH)D deficiency may be caused through abnormal nutrition transformation across placenta. Further, the suppression of the Nrf2/CBR1 pathway in maternal placenta also increases the possibility of MetS afflicted offspring.

Maternal 25(OH)D status around the time of conception has been found to be closely related to many health outcomes in mother and offspring. Numerous studies reported the associations between 25(OH)D status in pregnancy and a variety of obstetric complications, including gestational hypertension, pre-eclampsia, and gestational diabetes (GDM) (Curtis et al., 2018). Three separated meta-analyses of published studies all concluded that women with GDM had signiﬁcantly lower mean 25(OH)D than normal glycemic women (Poel et al., 2012; Aghajafari et al., 2013; Wei et al., 2013). Additionally, different sources have reported that a lower 25(OH)D status during pregnancy may induce other fetal and childhood outcomes, including adiposity and lean mass (Hanieh et al., 2014). Consistently, 25(OH)D deficiency in pregnant rats induces higher levels of FDG, FI, FDT, and HDL-C in both the liver and pancreas of the offspring, all of which are risks for developing MetS (**Figure 1**). These results highlight the critical role of 25(OH)D in MetS. Based on these findings, we examined the therapeutic action of 25(OH)D in cultured BRL cells. BRL cells cultured with 25(OH)D were found to have increased Nrf2/CBR1 pathway activation, as well as enhanced anti-oxidative functions (**Figure 6**). Therefore, we conclude that supplement of 25(OH)D maybe a therapy to alleviate metabolic disorders induced by oxidative stress. In addition, studies have demonstrated that Vitamin D could activate the Nrf2-Keap1 antioxidant pathway and improve renal disease in diabetic rats (Nakai et al., 2014); it can also activate the Nrf2 signaling pathway *via* vitamin D receptor to ameliorate leptin-induced oxidative stress (Teixeira et al., 2017). These results suggest that vitamin D receptor is an important component for vitamin D-induced activation of Nrf2 pathway. Therefore, we speculate vitamin D receptor might also involve in maternal 25(OH)D deficiency promoted MetS in offspring.

However, there were some limitations in our study that still need to be thoroughly investigated. First, because the details of metabolic disorder are extremely complex, the molecular mechanisms of metabolic disorder regulated by 25(OH)D should be examined outside of the role it plays in the Nrf2/CBR1 pathway. Also, considering Nrf2 is a transcription factor, further clarification is needed to determine if Nrf2 regulates the transcription of other antioxidant genes, in addition to CBR1. In addition, although our study unveiled the influence of maternal 25(OH)D deficiency on offspring, different degrees of influence on MetS may result from maternal 25(OH)D deficiency in different genders of offspring and a lack of glucose tolerance test is one limitation in our study. Hence, understanding how the gender of the offspring influences the response to maternal 25(OH)D deficiency still remains to be explored.

In conclusion, in our study, we find that maternal 25(OH)D deficiency is likely to result in offspring's MetS probably *via* abnormal nutrition transformation across placenta. Furthermore, the depression of the Nrf2/CBR1 pathway in both the mother and her offspring is one of the cause of oxidative stress leading to MetS. This study suggests that 25(OH)D treatment may relieve the offspring's Mets.

## Data Availability Statement

The raw data supporting the conclusions of this article will be made available by the authors, without undue reservation, to any qualified researcher.

## Ethics Statement

The animal study was reviewed and approved by the Ethics Committee of the Laboratory Animal Centre, Wenzhou Medical University.

## Author Contributions

HY and JQZ contributed to the study design. XL, BZ, ZZZ, and HZ collected data. JYZ, XX, and CS analyzed data. HC, JY, and ZW interpreted the results. ML, JC, QZ, ZZ, and JQZ wrote and revised the final draft of the manuscript.

## Funding

This work was supported by the Natural Science Foundation of Zhejiang Province under Grant No. LY13H040010, LQ18H040002.

## Conflict of Interest

The authors declare that the research was conducted in the absence of any commercial or financial relationships that could be construed as a potential conflict of interest.

## Supplementary Material

Supplementary Figure 1 Serum 25(OH)D concentrations in dams at on embryonic day 0 (E0) and embryonic day 21 (E21). Mean ± SD, n = 5 rat per group. **P* < 0.05, ***P* < 0.01, vs. control group. Normal group, Con; maternal 25-hydroxyvitamin D deficiency group, VDD.

Supplementary Figure 2 The bodyweights and the adjusted energy intake of the two groups from birth to 16 weeks in the offspring **(A**, **B)**. Mean ± SD, n = 5 rat per group. **P* < 0.05, ***P* < 0.01, vs. control group. Normal group, Con; maternal 25-hydroxyvitamin D deficiency group, VDD.

Supplementary Figure 3 Maternal 25-hydroxyvitamin D deficiency altered expression levels of HO-1, HQO1 and GCLC in offspring liver at week 0 and 16. ***P* < 0.01, **P* < 0.05, vs. control group. Normal group, Con; maternal 25-hydroxyvitamin D deficiency group, VDD.

Supplementary Figure 4 Immunohistochemistry-positive staining signals of IL-1β, IL-6 and TNFα were analyzed by Image J software. **P* < 0.05, ***P* <0.01, vs. control group. Normal group, Con; maternal 25-hydroxyvitamin D deficiency group, VDD.

Supplementary Figure 5 The affirmation of Nrf2 inhibitor (Brusatol) concentration and the siCBR1 availability. **(A)** BRL cells were treated with Nrf2 inhibitor Brusatol at 0, 20, 40, 80, and 160 nM. The results from Western blot demonstrates the effect of Brusatol at various concentration on Nrf2 expression level. **(B)** Western blot analysis was performed to examine the protein levels of CBR1 in control (scrambled) siRNA- and CBR1-specific siRNA using different siRNA (siCBR1-1, siCBR1-2, siCBR1-3)-transfected BRL cells. Densitometry data for CBR1 from the blots shown were normalized for analysis to GAPDH. The results are expressed as mean ± SD of three independent determinations. ***P* < 0.01, ****P* < 0.001, vs. control group. Normal group, untreated group, Con; siCBR1-1, siC-1; siCBR1-2, siC-2; siCBR1-3, siC-3.

## Abbreviations

MetS: metabolic syndrome; IR: insulin resistance; Nrf2: nuclear factor erythroid 2 related factor 2; CBR1: carbonyl reductase 1; GAPDH: glyceraldehyde-3-phosphate dehydrogenase; qRT-PCR: quantitative real-time polymerase chain reaction; siRNA: small interfering RNA; TG: triglyceride; HDL-C: high-density lipoprotein cholesterol; INS: insulin; FBG: fasting blood glucose; GDM, gestational diabetes; FBS: fetal bovine serum; PBS: phosphate-buffered solution.
