# Supplementary figures and images for: Maternal 25-Hydroxyvitamin D Deficiency Promoted Metabolic Syndrome and Downregulated Nrf2/CBR1 Pathway in Offspring
Source: Front Pharmacol. 2020 Feb 28;11:97. doi: 10.3389/fphar.2020.00097 (PMC7058637; doi:10.3389/fphar.2020.00097)

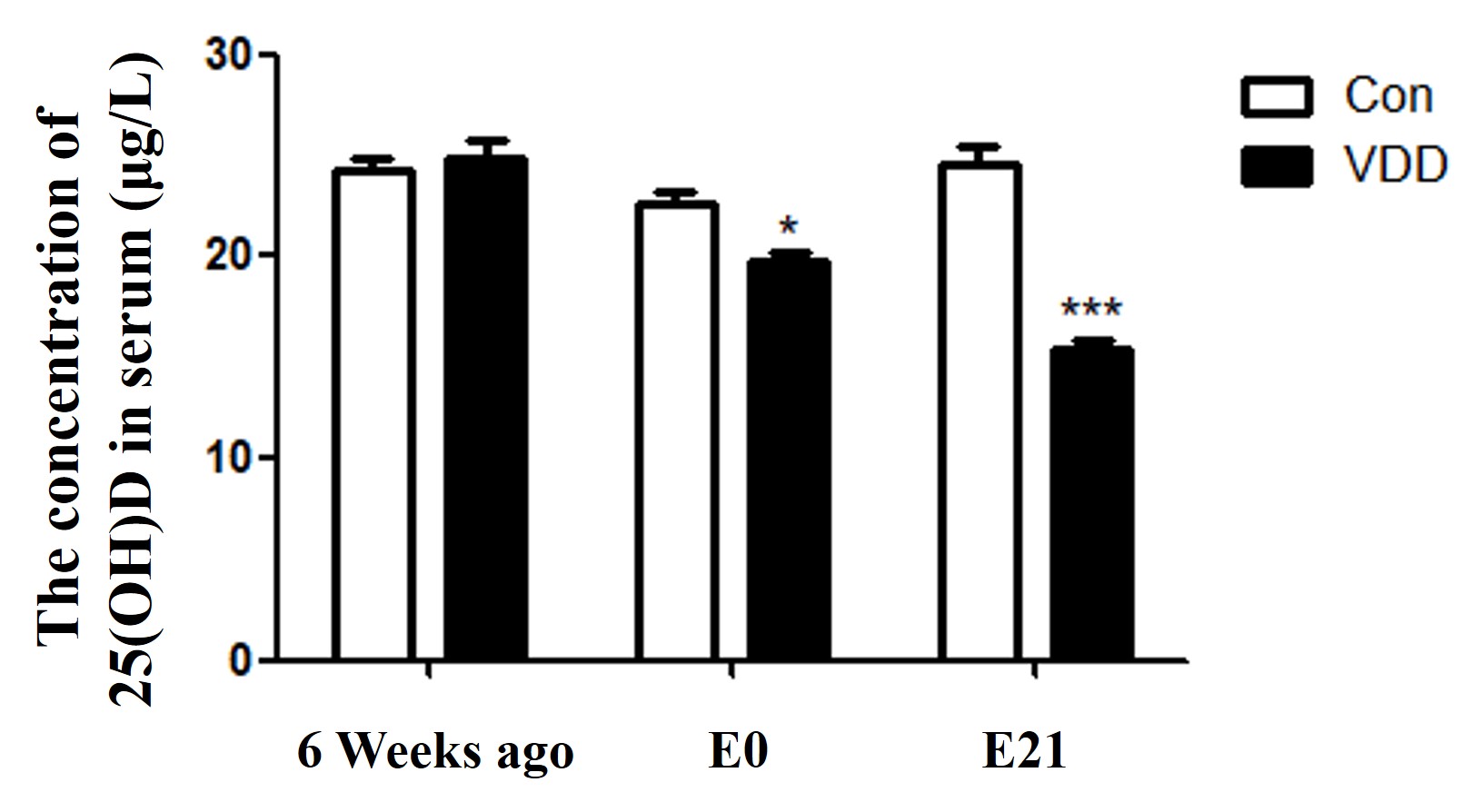

Supplement: Supplementary Figure 1 — Serum 25(OH)D concentrations in dams at on embryonic day 0 (E0) and embryonic day 21 (E21). Mean ± SD, n = 5 rat per group. *P < 0.05, **P < 0.01, vs. control group. Normal group, Con; maternal 25-hydroxyvitamin D deficiency group, VDD. [file Image_1.jpeg]

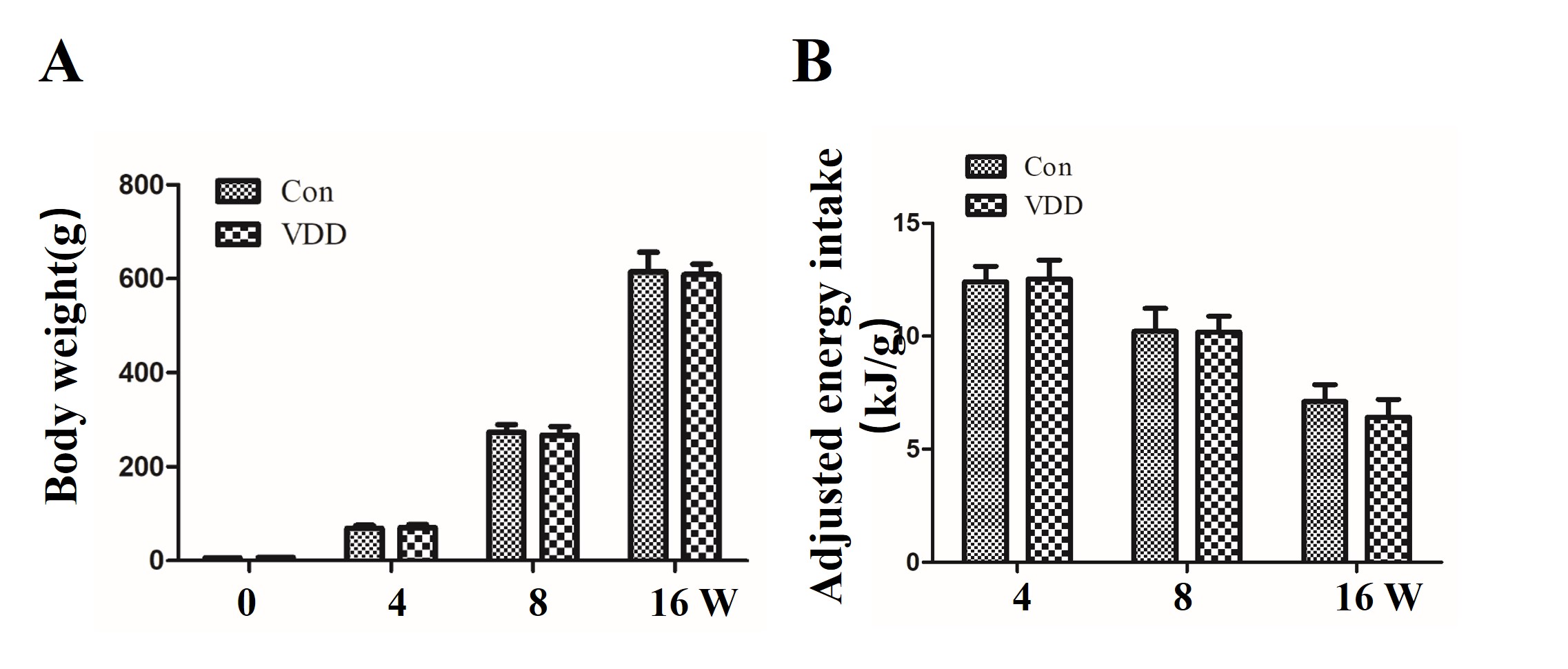

Supplement: Supplementary Figure 2 — The bodyweights and the adjusted energy intake of the two groups from birth to 16 weeks in the offspring (A, B). Mean ± SD, n = 5 rat per group. *P < 0.05, **P < 0.01, vs. control group. Normal group, Con; maternal 25-hydroxyvitamin D deficiency group, VDD. [file Image_2.jpeg]

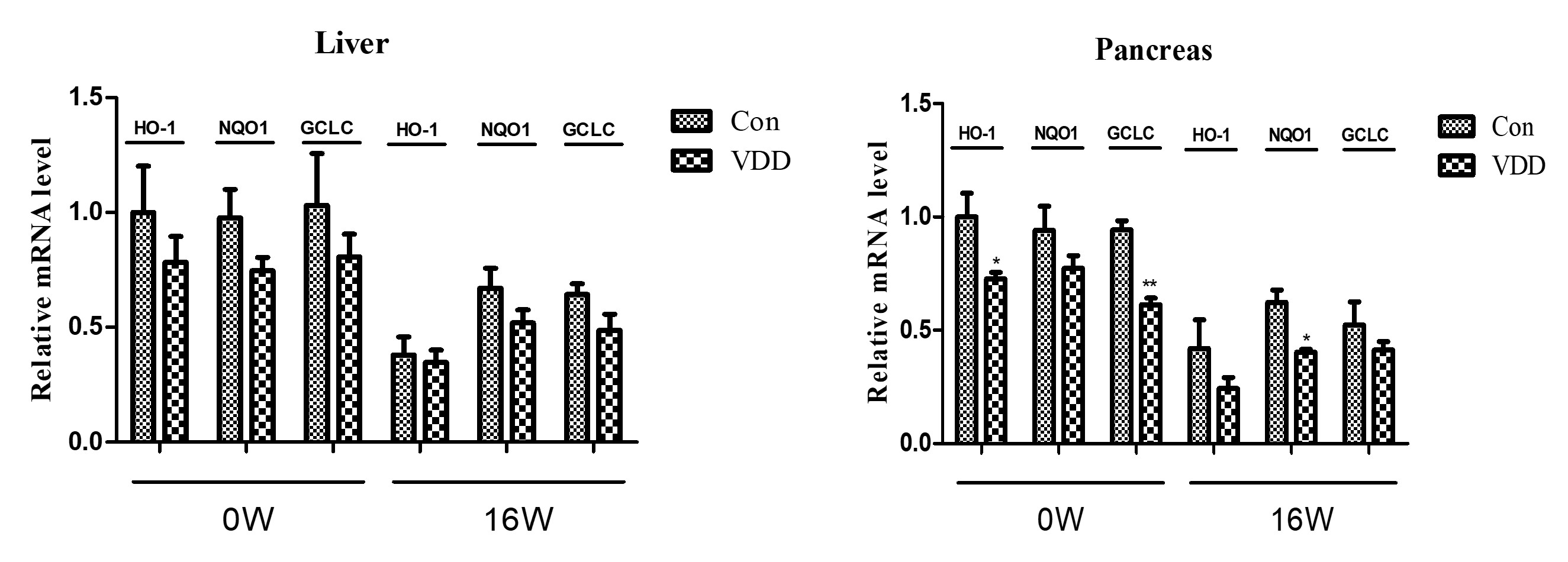

Supplement: Supplementary Figure 3 — Maternal 25-hydroxyvitamin D deficiency altered expression levels of HO-1, HQO1 and GCLC in offspring liver at week 0 and 16. **P < 0.01, *P < 0.05, vs. control group. Normal group, Con; maternal 25-hydroxyvitamin D deficiency group, VDD. [file Image_3.jpeg]

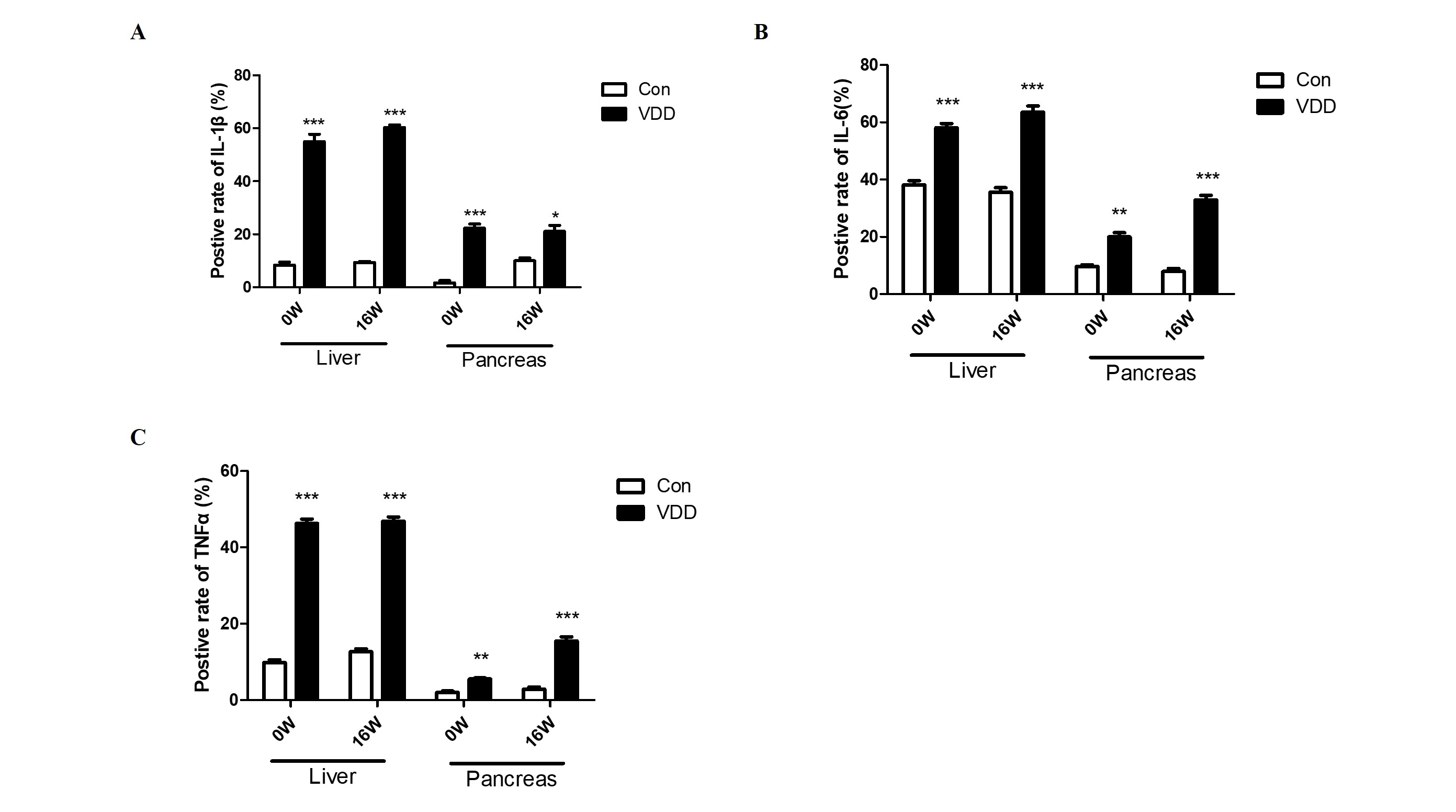

Supplement: Supplementary Figure 4 — Immunohistochemistry-positive staining signals of IL-1β, IL-6 and TNFα were analyzed by Image J software. *P < 0.05, **P <0.01, vs. control group. Normal group, Con; maternal 25-hydroxyvitamin D deficiency group, VDD. [file Image_4.jpeg]

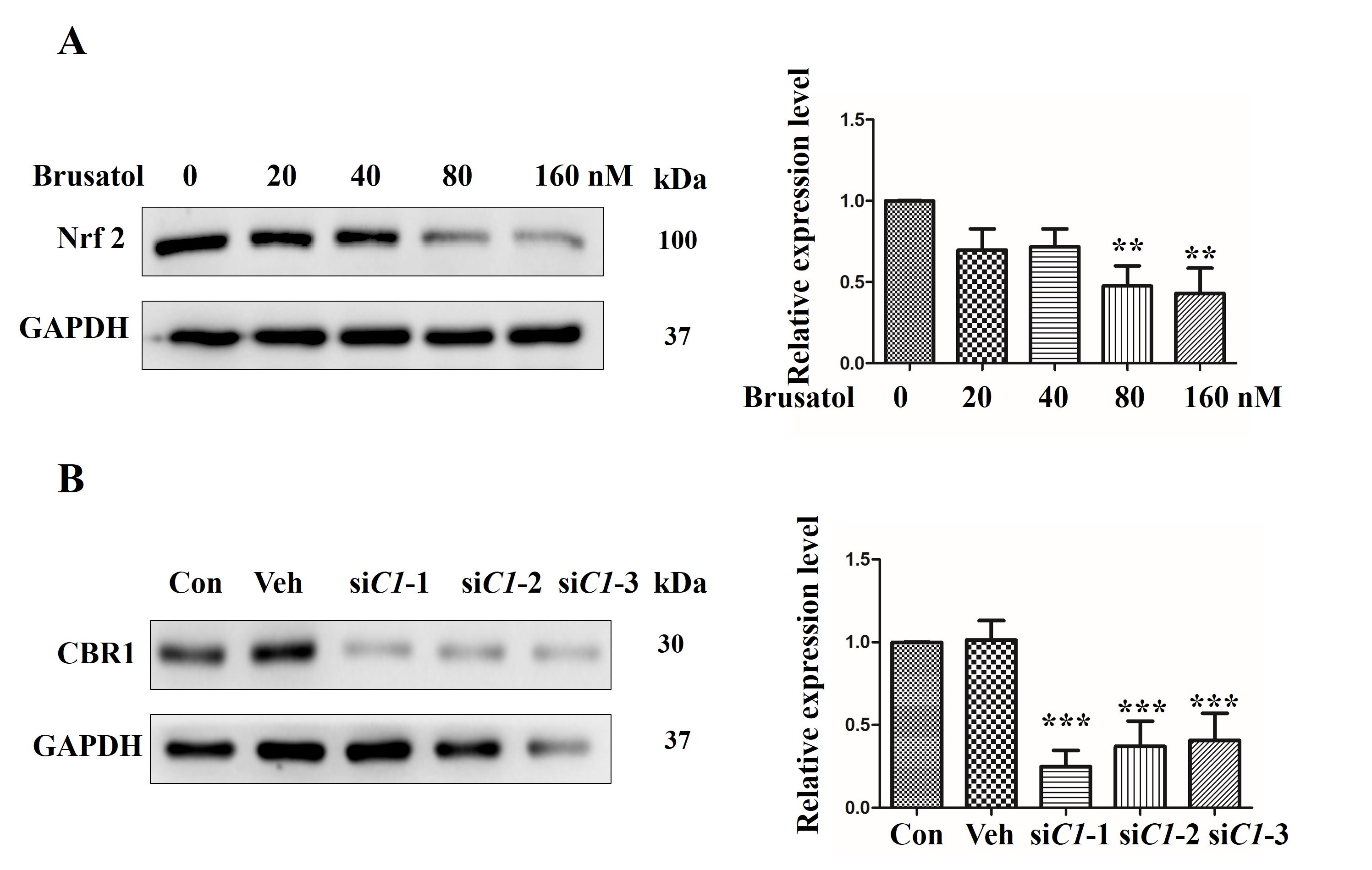

Supplement: Supplementary Figure 5 — The affirmation of Nrf2 inhibitor (Brusatol) concentration and the siCBR1 availability. (A) BRL cells were treated with Nrf2 inhibitor Brusatol at 0, 20, 40, 80, and 160 nM. The results from Western blot demonstrates the effect of Brusatol at various concentration on Nrf2 expression level. (B) Western blot analysis was performed to examine the protein levels of CBR1 in control (scrambled) siRNA- and CBR1-specific siRNA using different siRNA (siCBR1-1, siCBR1-2, siCBR1-3)-transfected BRL cells. Densitometry data for CBR1 from the blots shown were normalized for analysis to GAPDH. The results are expressed as mean ± SD of three independent determinations. **P < 0.01, ***P < 0.001, vs. control group. Normal group, untreated group, Con; siCBR1-1, siC-1; siCBR1-2, siC-2; siCBR1-3, siC-3. [file Image_5.jpeg]
